# Time of day influences stress hormone response to ketamine

**DOI:** 10.1111/jne.13194

**Published:** 2022-09-02

**Authors:** Matthew T. Birnie, Alen V. Eapen, Yvonne M. Kershaw, David Lodge, Graham L. Collingridge, Becky L. Conway‐Campbell, Stafford L. Lightman

**Affiliations:** ^1^ Henry Wellcome Laboratories for Integrative Neuroendocrinology, School of Medicine University of Bristol Bristol UK; ^2^ School of Physiology, Pharmacology & Neuroscience University of Bristol Bristol UK

**Keywords:** circadian, cortisol/corticosterone, glucocorticoids, ketamine

## Abstract

Over 50% of depressed patients show hyperactivity of the hypothalamic–pituitary–adrenal (HPA) axis. Conventional therapy takes weeks to months to improve symptoms. Ketamine has rapid onset antidepressant effects. Yet its action on HPA axis activity is poorly understood. Here, we measured the corticosterone (CORT) response to ketamine administered at different times of day in the Wistar–Kyoto (WKY) rat. In male rats, blood was collected every 10 min for 28 h using an automated blood sampling system. Ketamine (5/10/25 mg · kg) was infused through a subcutaneous cannula at two time points–during the active and inactive period. CORT levels in blood were measured in response to ketamine using a radioimmunoassay. WKY rats displayed robust circadian secretion of corticosterone and was not overly different to Sprague Dawley rats. Ketamine (all doses) significantly increased CORT response at both infusion times. However, a dose dependent effect and marked increase over baseline was observed when ketamine was administered during the inactive phase. Ketamine has a robust and rapid effect on HPA axis function. The timing of ketamine injection may prove crucial for glucocorticoid‐mediated action in depression.

## Graphical abstract

Over 50% of depressed patients show hyperactivity of the HPA axis. Ketamine has rapid onset antidepressant effects, yet how ketamine interacts with HPA axis function is unknown. Here, we measured corticosterone release in the rat across the day and during ketamine infusion in the morning and evening. We found that Ketamine infused during the morning induced a greater corticosterone response than in the evening. These data suggest that time of day may be crucial for glucocorticoid‐mediated action in depression.

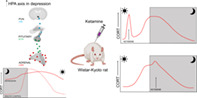

## 1 INTRODUCTION

Major depressive disorder (MDD) is a leading cause of the burden of disease globally.^1^ It is frequently characterized by abnormal neurotransmitter function,^2^ for instance serotonin, norepinephrine, and dopamine—evidenced by using wide‐ranging antidepressants including selective serotonin reuptake inhibitors (SSRIs), serotonin and norepinephrine reuptake inhibitors (SNRIs) and norepinephrine and dopamine reuptake inhibitors (NDRIs).^3^, ^4^, ^5^ However, these treatments are often unsuccessful,^6^ identifying an urgent need to understand the neurobiology underpinning MDD and to discover effective treatment strategies.^7^, ^8^

The hypothalamic–pituitary–adrenal (HPA) axis synthesizes and releases the steroid hormone cortisol (human) and corticosterone (rodent).^9^ Dysregulated HPA axis function, marked by abnormal CORT secretion and responses to stress, are hallmarks of several chronic conditions, as well as MDD.^10^, ^11^, ^12^ Endogenous models of MDD, such as the Wistar–Kyoto (WKY) rat^13^ provide valuable insights for investigating the pathophysiology of MDD. Originally bred as normative controls for the spontaneous hypertensive rat, they display behavioral and neurobiological phenotypes like those observed in clinical cases of MDD, such as learned helplessness, stress‐induced ulcers, and reported elevated glucocorticoids,^14^, ^15^, ^16^ as well as resistance to common antidepressants.^17^

Ketamine has long been used in clinical practice,^18^, ^19^ progressing from an anesthetic induction agent to being used in the treatment of therapy resistant major depression.^20^, ^21^ Although its use and action on HPA axis function have previously been studied in animals and humans,^22^, ^23^ results have often been ambiguous, and less is known about ketamine's action on HPA axis function at different times of day.

In this study, we firstly characterize the WKY rat endogenous circulating CORT profile and follow up with distinguishing the complex action and potential importance of time‐of‐day administration of ketamine for HPA axis responsiveness.

## 2 METHODS

Male Sprague Dawley (SD) and WKY rats (250 g, ~3 months old; Envigo, UK) were used in experimental procedures. Animals were maintained in soundproof rooms in standard housing conditions under a 14:10 light/dark cycle. Food and water were available ad libitum. All procedures were carried out in accordance with the UK Animals (Scientific Procedures) Act 1986 under PPL 30/3114.

Rats were anaesthetized with a combination of Isoflurane (100% w/w liquid vapor [Merial, UK]) and medical air during right jugular vein cannulation and subcutaneous cannula placement. The cannula (Smith Medicals, UK) was exteriorized via a vascular access button and attached to a spring and swivel system. Postoperative analgesic 1 mg of carprofen (Rimadyl, Pfizer, UK), and 2.5 ml of glucose (5%)/saline (0.9%) were administered subcutaneously. Implanted cannulae were “flushed” daily and replaced with fresh heparinized saline (1:100) to maintain patency. All rats were given 5 days post‐surgical recovery prior to subcutaneous infusion of ketamine.

### 2.1 Automated blood sampling

Blood samples were collected via an in‐house automated blood sampler. Forty microliters blood samples were collected every 10 min for 28 h in 160 μl heparin‐saline (1:100). Plasma was separated from whole blood by centrifugation at 4000 rpm, 4°C and diluted 1 in 50 with citrate buffer, processed in triplicate and incubated overnight in 50 μl of ^125^I corticosterone tracer (Oxford BioInnovation DSL Ltd, Oxford, UK) with 50 μl of rabbit anti‐rat corticosterone antibody (kindly donated by G. Makara, Hungary). Free/bound separation was performed using charcoal dextran precipitation and centrifuged pellets ^125^I corticosterone levels were measured using a gamma counter (Wizard‐2470, Perkin Elmer, MA). Concentrations of corticosterone in each plasma sample were interpolated from a standard curve. Blood volume was replaced with heparin‐saline during each sampling period. We have previously shown in many publications that this is not associated with any change in HPA activity, although it does result in a small fall in hematocrit.

### 2.2 Automated ketamine infusion

Ketamine (5/10/25 mg · kg) (Ketalar; ketamine hydrochloride) or vehicle (0.9% saline) was infused at two different times of day (AM–Zeitgeber 2; PM–Zeitgeber 16) as a bolus subcutaneous infusion (10 min) using a programmable pump (PHD Ultra Syringe Pump, Harvard Apparatus, USA).

### 2.3 Statistical analysis

**FIGURE 1:**
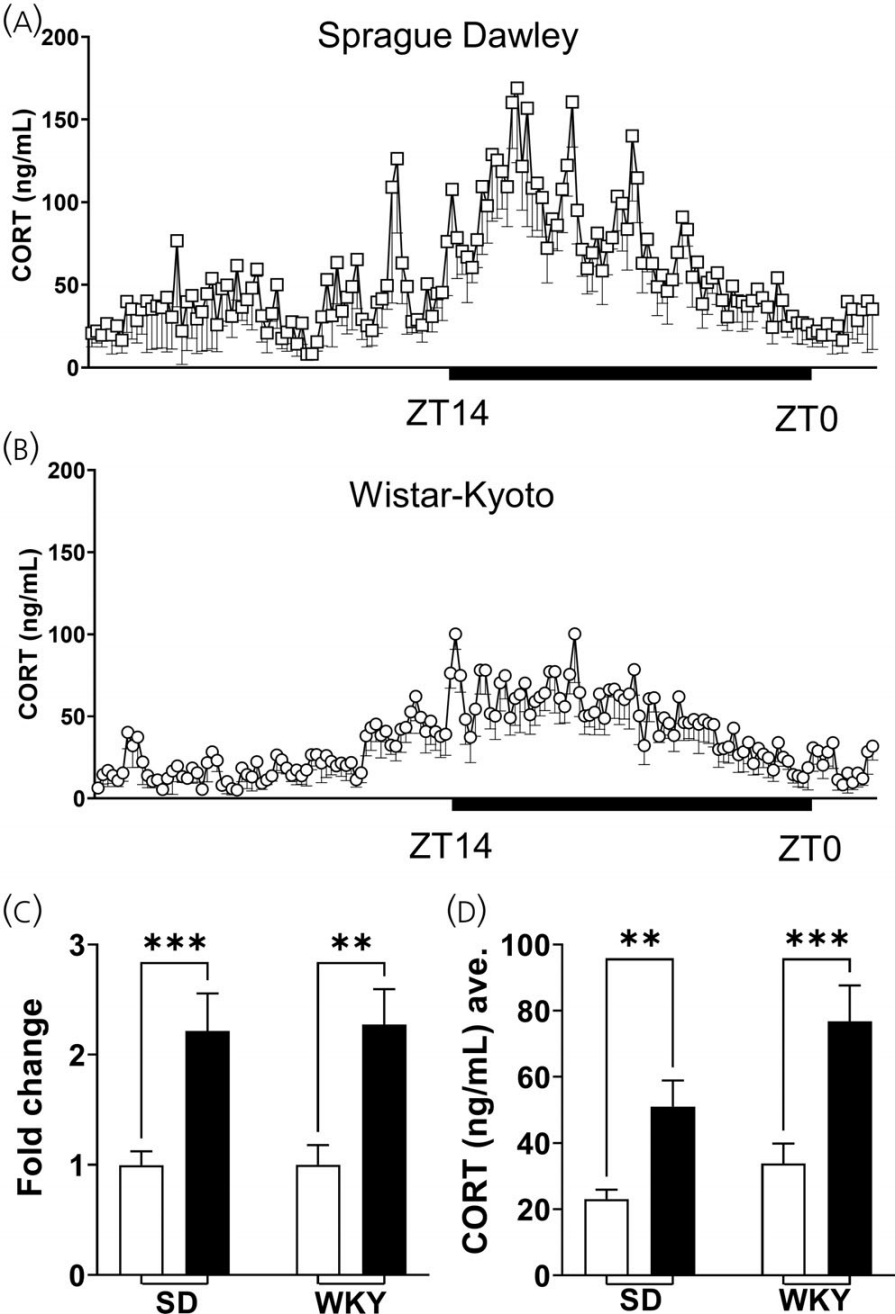
Endogenous circadian corticosterone (CORT) secretion is maintained in a rat model of depression. (A) Twenty‐eight hours of CORT secretion in SD rats. (B) Twenty‐eight hours of CORT secretion in WKY rats. (C) Two‐way RM‐ANOVA of fold change in CORT secretion comparing strain and time of day (light/dark) showed a significant effect of time (*F*_1,10_ = 49.25, *p* < .0001, Sidak's post hoc–SD; *p* = .0007; WKY–*p* = .0017), but not strain (*F*_1,10_ = .008, *p* = .9291) nor interaction (*F*_1,10_ = .023, *p* = .8802). (D) Two‐way RM‐ANOVA of average CORT secretion comparing strain and light and dark periods showed an effect of time (*F*_1,10_ = 54.38, *p* < .0001, Sidak's post hoc—SD; *p* = .0023; WKY—*p* = .0003), a trend for strain (*F*_1,10_ = 4.132, *p* = .0695) but no interaction (*F*_1,10_ = 2.42, *p* = .1509). Data are represented as mean ± SEM of 5–7 rats. Black bars indicate period of dark/active period in 24 h cycle. RM‐ANOVA, repeated measures‐analyses of variance; SD, Sprague–Dawley; WKY, Wistar–Kyoto

**FIGURE 2:**
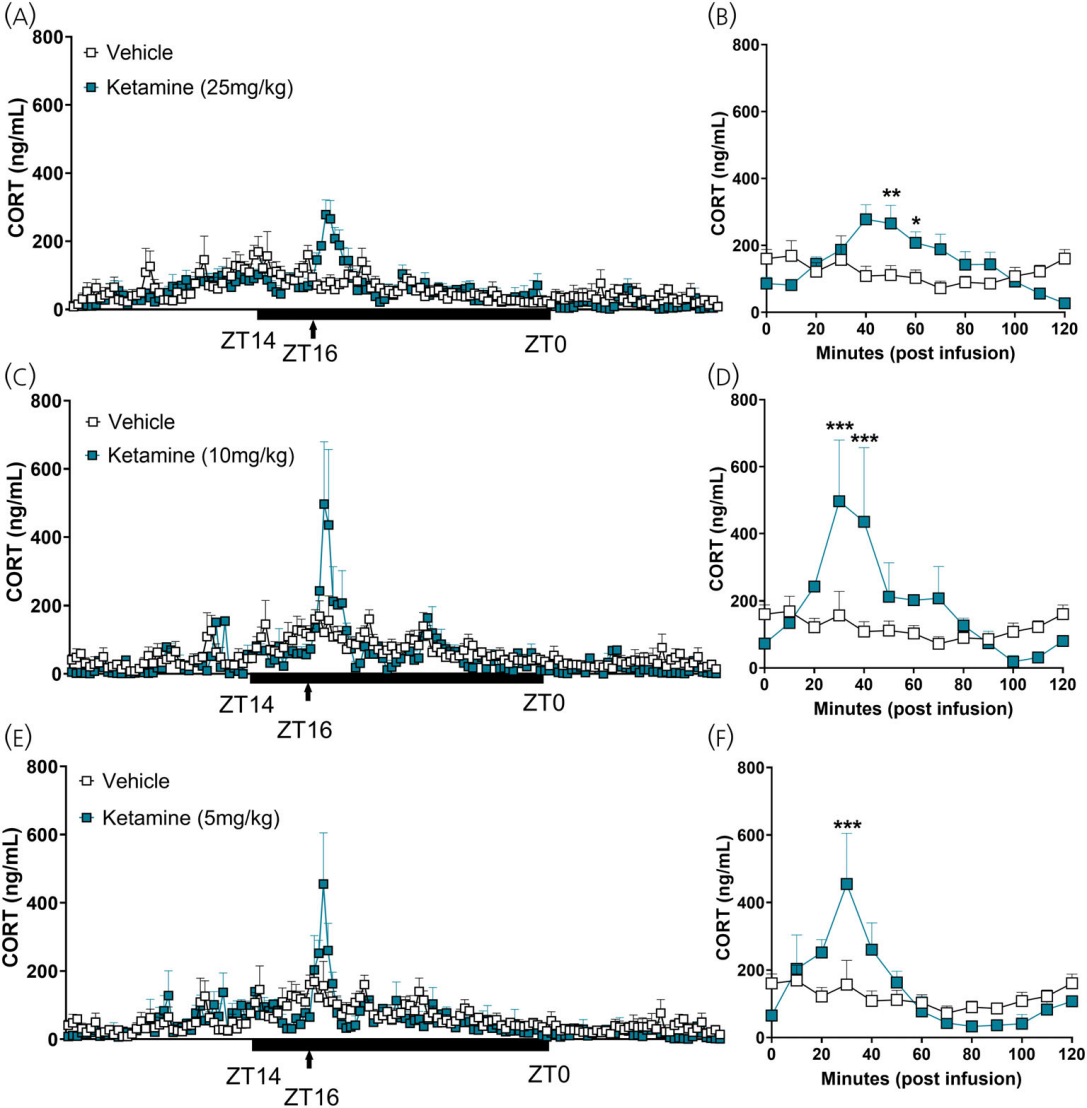
Effect of ketamine on corticosterone secretion infused during the active period. (A) Automated blood sampling for 28 h of CORT secretion in WKY rats with a time automated infusion of ketamine (25 mg · kg) during the active period (at zeitgeber 16). Mixed effects analysis identified a significant effect of time (*F*_5.728,37.53_ = 7.094, *p* < .0001), no effect of treatment (*F*_1,8_ = .071, *p* = .7966), but an interaction (*F*_143,937_ = 2.51, *p* < .0001). (B) Corticosterone levels for 120 min following ketamine (25 mg · kg) infusion. Two‐way ANOVA reported a significant effect of time (*F*_12,96_ = 2.103, *p* = .0235), a trend to significance with treatment (*F*_1,96_ = 3.730, *p* = .0564) and an interaction (*F*_12,96_ = 4.065, *p* < .0001, Sidak's post hoc: 50 min: *p* = .0046; 60 min: *p* = .0139). (C) Twenty‐eight hours of CORT secretion in WKY rats with an infusion of ketamine (10 mg · kg) during the active period. Mixed effects analysis detected a significant effect of time (*F*_3.208,12.03_ = 5.176, *p* = .0148), no effect of treatment (*F*_1,6_ = .4216, *p* = .5402), but an interaction (*F*_143,536_ = 1.769, *p* < .0001). (D) Corticosterone levels for 120 min following ketamine (10 mg · kg) infusion. Two‐way ANOVA found a significant effect of time (*F*_12,60_ = 3.956, *p* = .0002), treatment (*F*_1,60_ = 6.612, *p* = .0126) and an interaction (*F*_12,60_ = 3.603, *p* = .0005, Sidak's post hoc: 30 min: *p* = .0004; 40 min: *p* = .0007). (E) Twenty‐eight hours of CORT secretion in WKY rats with an infusion of ketamine (5 mg · kg) during the active period. Mixed effects analysis found a significant effect of time (*F*_3.874,18.88_ = 5.972, *p* = 0.003), no effect of treatment (*F*_1,7_ = 0.3886, *p* = .5528), but an interaction (*F*_143,697_ = 1.814, *p* < .0001). (F) Corticosterone levels for 120 min following ketamine (5 mg · kg) infusion. Two‐way ANOVA reported a significant effect of time (*F*_12,79_ = 4.894, *p* < .0001), no effect of treatment (*F*_1,79_ = 1.03, *p* = .3132) but a significant interaction (*F*_12,79_ = 2.987, *p* = 0.0017, Sidak's post hoc: 30 min: *p* = .0001). Data are represented as mean ± SEM of 3–5 rats per treatment. Black bars indicate period of dark/active period in 24 h cycle. ANOVA, repeated measures‐analyses of variance; WKY, Wistar–Kyoto

**FIGURE 3:**
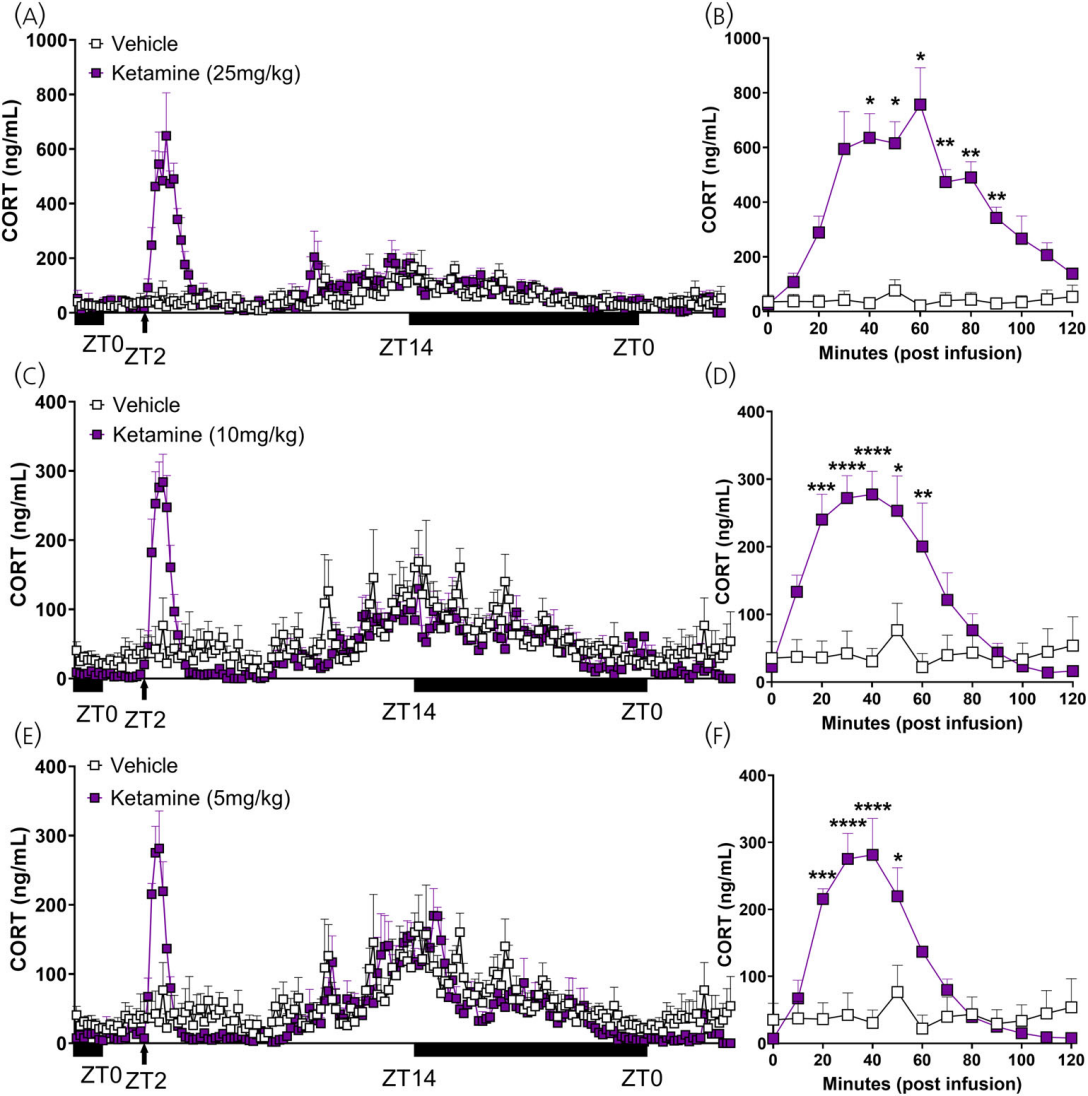
Effect of ketamine on corticosterone secretion infused during the inactive period. (A) Automated blood sampling for 28 h measures CORT secretion in WKY rats with a time‐automated infusion of ketamine (25 mg · kg) during the inactive period (at zeitgeber 2). Mixed effects analysis identified a significant effect of time (*F*_3.893,32.62_ = 5.177, *p* = .0026), treatment (*F*_1,10_ = 6.564, *p* = .0283), and an interaction (*F*_174,1458_ = 3.838, *p* < .0001). (B) Corticosterone levels for 120 min following ketamine (25 mg · kg) infusion. Mixed effects analysis detected a significant effect of time (*F*_3.495,22.72_ = 5.936, *p* = .0027), treatment (*F*_1,9_ = 37.87, *p* = .0002) and an interaction (*F*_12,78_ = 6.218, *p* < 0.0001, Sidak's post hoc: 40 min: *p* = .0105; 50 min: *p* = .0217; 60 min: *p* = .0334; 70 min: *p* = .0084; 80 min: *p* = .0027; 90 min: *p* = .0015). (C) Twenty‐eight hours of CORT secretion in WKY rats with an infusion of ketamine (10 mg · kg) during the inactive period. Mixed effects analysis detected a significant effect of time (*F*_3.673,18.22_ = 6.159, *p* = .003), no effect of treatment (*F*_1,7_ = .3057, *p* = .5975), but an interaction (*F*_174,863_ = 2.771, *p* < .0001). (D) Corticosterone levels for 120 min following ketamine (10 mg · kg) infusion. Two‐way ANOVA reported a significant effect of time (*F*_12,61_ = 5.336, *p* < .0001), treatment (*F*_1,61_ = 47.15, *p* < .0001) and an interaction (*F*_12,61_ = 5.172, *p* < .0001, Sidak's post hoc: 20 min: *p* = .0004; 30 min: *p* < .0001; 40 min: *p* < .0001; 50 min: *p* = .0136; 60 min: *p* = .0028). (E) Twenty‐eight hours of CORT secretion in WKY rats with an infusion of ketamine (5 mg · kg) during the inactive period. Comparably to infusion with 10 mg · kg ketamine, mixed effects analysis detected a significant effect of time (*F*_3.743,17.25_ = 6.701, *p* = .0022), no effect of treatment (*F*_1,7_ = .3262, *p* = .5857), but an interaction (*F*_174,802_ = 2.170, *p* < .0001). (F) Corticosterone levels for 120 min following ketamine (5 mg · kg) infusion. Two‐way ANOVA reported a significant effect of time (*F*_12,48_ = 7.241, *p* < .0001), treatment (*F*_1,48_ = 32.79, *p* < .0001) and an interaction (*F*_12,48_ = 6.940, *p* < .0001, Sidak's post hoc: 20 min: *p* = .0004; 30 min: *p* < .0001; 40 min: *p* < .0001; 50 min: *p* = .0143). Data are represented as mean ± SEM of 4–7 rats per treatment. Black bars indicate period of dark/active period in 24 h cycle. ANOVA, repeated measures‐analyses of variance; RM‐ANOVA, repeated measures‐ANOVA; WKY, Wistar–Kyoto

**FIGURE 4:**
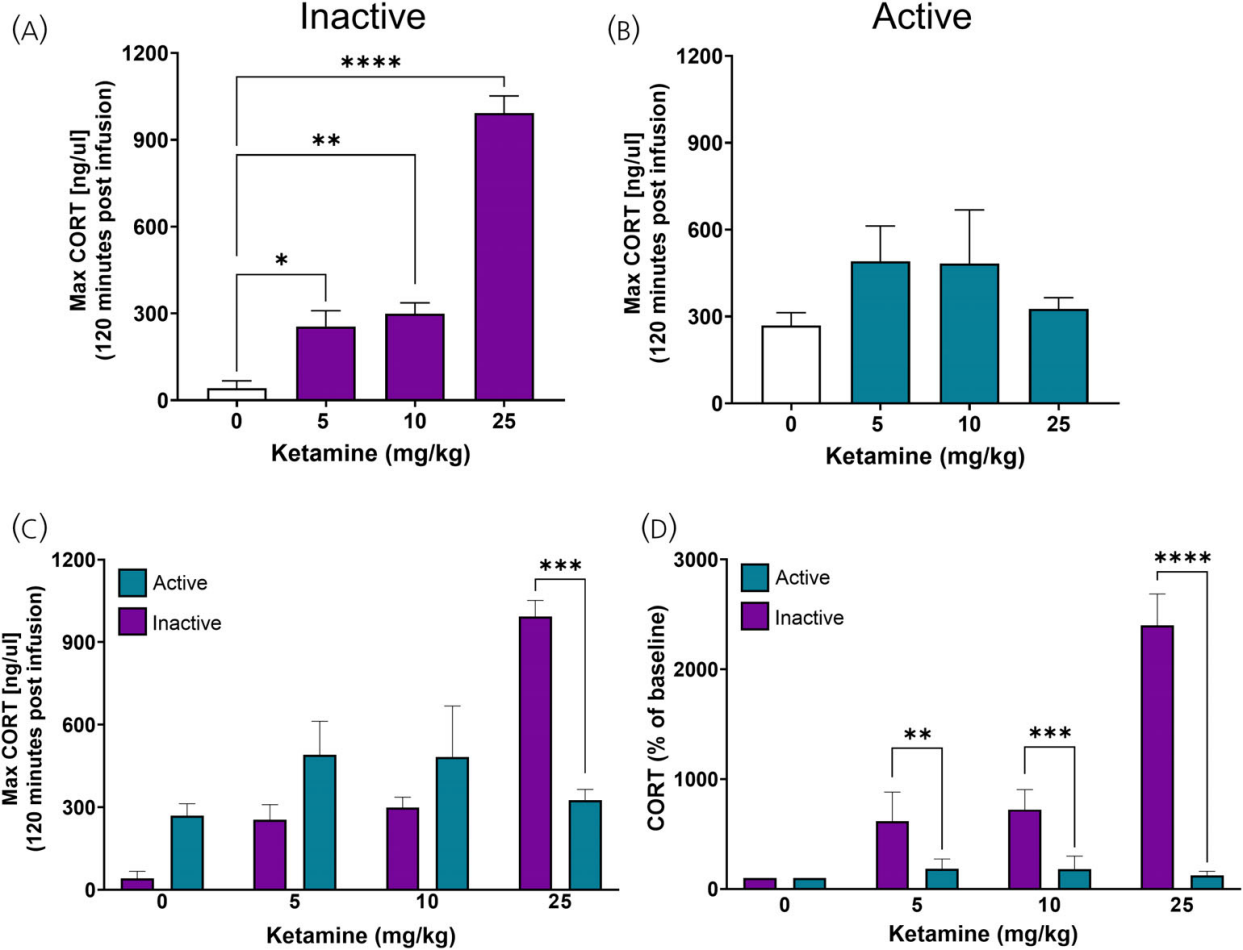
Time of day influences corticosterone response to ketamine dose. (A) Maximal CORT secretion following ketamine infusion (all doses) during the inactive period. One‐way ANOVA reported a significant effect of ketamine infusion on CORT secretion compared to vehicle infusion (*F*_3,12_ = 78.89, *p* < .0001, Dunnett's post hoc comparisons: 5 mg · kg: *p* = .0182; 10 mg · kg: *p* = .0055; 25 mg · kg: *p* < .0001). (B) Maximal CORT secretion following ketamine infusion (all doses) during the active period. One‐way ANOVA did not find an effect of ketamine infusion on CORT secretion compared to vehicle infusion (*F*_3,13_ = 1.489, *p* = 0.2637). (C) Maximal CORT secretion during active and inactive periods following ketamine infusion (all doses). Mixed effects analysis did not identify a significant effect of time (*F*_1,11_ = .008, *p* = .9307), but did identify a significant effect of treatment (*F*_3,14_ = 16.71, *p* < .0001) and an interaction (*F*_3,11_ = 19.24, *p* = .0001, Sidak's post hoc analysis: 25 mg · kg: *p* = .0001). (D) CORT secretion as a % of vehicle peak levels following ketamine infusion (all doses). Mixed effects analysis identified a significant effect of time (*F*_1,25_ = 216.6, *p* < .0001), treatment (*F*_3,25_ = 85.53, *p* < .0001) and an interaction (*F*_3,25_ = 85.74, *p* < .0001, Sidak's post hoc analysis: 5 mg · kg: *p* = .0025; 10 mg · kg: *p* = .0005; 25 mg · kg: *p* < .0001). Data are represented as mean ± SEM of 3–7 rats per treatment. ANOVA, repeated measures‐analyses of variance

Results are presented as mean ± SEM. All statistical analyses were performed using GraphPad Prism (version 9.1, GraphPad Software), using restricted maximum likelihood (REML) mixed effects analyses, analyses of variance (ANOVA) or repeated measures ANOVA (RM ANOVA), followed by post hoc tests where appropriate. Two‐way‐RM ANOVA was used to test if differences in corticosterone secretion were observed across time and in two different rat strains—SD and Wistar Kyoto (Figure 1). In A, C, and E (Figures 2 and 3), mixed effects analysis was used to assess if ketamine infusion affected corticosterone secretion across time. With automated blood sampling, samples being collected every 10 min can result in a poor sample draw. This prevented the use of two‐way‐RM ANOVA. In B, D, and F (Figures 2 and 3), two‐way‐RM ANOVA was used to identify if ketamine infusion affected corticosterone secretion across time immediately following infusion. In A and B (Figure 4), one‐way ANOVA was used to compare the peak level of corticosterone within 120 min of ketamine infusion during the inactive and active periods, respectively. In A, Dunnett's post hoc test was used to compare the peak levels to a control (vehicle) group. In C and D, mixed effects analysis (REML) was used to compare the effect of ketamine dose across time. Sidak's post hoc test was used to compare effect of drug within time. Details of specific analyses are provided in the text and figure legends. Statistical significance was set at *p* < .05.

## 3 RESULTS

### 3.1 Characterization of endogenous corticosterone secretion in SD and WKY rats

Endogenous CORT secretion exhibits a circadian and ultradian pattern of secretion. In rodents, CORT levels are low during the inactive phase (light phase), and peak upon wakening (active phase; dark phase).^24^ The WKY rat is used as an experimental model for depression,^13^ often characterized by altered HPA axis regulation. To assess if the WKY rat HPA axis function is different to that of the commonly used SD rat, blood was sampled every 10 min for 28 h. Mean plasma CORT concentration of SD (Figure 1A)—nondepressive‐like model, and the WKY rat (Figure 1B), are shown. Perhaps unsurprisingly, the WKY rat displays normal HPA axis function, with clear circadian and ultradian rhythm. However, to assess if there are differences in the circadian secretion of CORT between strains, epochs of active (dark phase) and inactive phases (light phase) were measured (Figure 1C). We show a clear increase in CORT in both SD and WKY strains—we report a main effect of time (*F*_1,10_ = 49.25, *p* < .0001: SD: *p* = .0007; WKY: *p* = .0017). Yet, to address the potential elevation in circulating CORT as a characteristic of the WKY rat, we measured average CORT secretion between active and inactive phases (Figure 1D). We found a main effect of time (*F*_1,10_ = 54.38, *p* < .0001: SD: *p* = .0023; WKY: *p* = .0003), but only a trend toward differences in strain (*F*_1,10_ = 4.132, *p* = .0695). This is interesting, as although the data did not reach significance, the elevated circulating CORT shown in the WKY rat may contribute to the endogenous phenotype it possesses. The WKY rat shows similar behavioral and neurobiological phenotypes to depression.^13^ We therefore continued with this endogenous model to identify the action of ketamine at different doses on HPA axis activity.

### 3.2 CORT response to ketamine infusion during the active phase in WKY rats

Comparable to above, we measured endogenous CORT secretion over 28 h in WKY rats. In addition, these rats also received a bolus (10 min) subcutaneous infusion of ketamine (5/10/25 mg · kg) during the active phase—in line with clinical treatment times. Importantly, infusion of vehicle did not elicit a CORT response. However, ketamine infusion increased CORT secretion rapidly with all doses (Figure 2A,C,E). Peak levels were not dose‐dependent; however, the maintenance of circulating CORT was different across doses. We measured the levels of circulating CORT for 120 min post infusion. A high dose of ketamine (25 mg · kg) delayed the increase in CORT secretion above vehicle until 50 min post infusion, before returning to baseline by 70 min (Figure 2B—25 mg · kg: Time; *F*_12,96_ = 2.103, *p =* .0235: Treatment; *F*_1,96_ = 3.73, *p =* .0564: Interaction; *F*_12,96_ = 4.065, *p <* .0001, 50 min: *p* = .0045, 60 min; *p* = .0139). Surprisingly, ketamine (10 mg · kg) increased CORT levels above vehicle faster than the higher dose (30 min), and was back to baseline by 50 min (Figure 2D—10 mg · kg: Time; *F*_12,60_ = 3.956, *p =* .0002: Treatment; *F*_1,60_ = 6.612, *p =* .0126: Interaction; *F*_12,60_ = 3.603, *p =* .0005, 30 min: *p* = .0004, 40 min: *p* = .0007). This effect was also evident with ketamine (5 mg · kg), where an increase in CORT secretion was observed only at 30 min (Figure 2F—5 mg · kg: Time; *F*_12,79_ = 4.894, *p <* .0001: Treatment; *F*_1,79_ = 1.03, *p =* .3132: Interaction; *F*_12,79_ = 2.987, *p =* .0017, 30 min: *p* = .0001). These data indicate a dose dependent effect on the maintenance of circulating glucocorticoids and suggest a differential timing of activity on the glucocorticoid receptor (GR) with ketamine treatment during the active period.

### 3.3 CORT responses to ketamine infusions during the inactive phase in WKY rats

In addition to dose, the timing of ketamine delivery may prove critical for the regulation and activity of HPA axis function.^25^ For this, we assessed the response to the same doses of ketamine but this time during the inactive period (when endogenous circulating glucocorticoids are low). Again, importantly, and similar to above, infusion of vehicle did not induce a CORT response. However, ketamine increased CORT rapidly, maintained levels for an extended period and resulted in a dose dependent increase (Figure 3A,C,E). Interestingly, the CORT response was not only faster, but was maintained longer when ketamine (25 mg · kg) when infused during this period—increasing CORT above vehicle at 40 min, and not returning to baseline until 90 min (Figure 3B—25 mg · kg: Time; *F*_3.495,22.72_ = 5.936, *p =* .0027: Treatment; *F*_1,9_ = 37.87, *p =* .0002: Interaction; *F*_12,78_ = 6.218, *p <* .0001, 40 min: *p* = .0105, 50 min: *p* = .0217, 60 min: *p* = .0334, 70 min: *p* = .0084, 80 min: *p* = .0027, 90 min: *p* = .0015). This was also evident with ketamine (10 mg · kg) (Figure 3D—10 mg · kg: Time; *F*_12,61_ = 5.336, *p <* .0001: Treatment; *F*_1,61_ = 47.15, *p <* .0001: Interaction; *F*_12,61_ = 5.172, *p <* .0001, 20 min: *p* = .0004, 30 min: *p* < .0001, 40 min: *p* < .0001, 50 min: *p* = .0136, 60 min: *p* = .0028) and (5 mg · kg) (Figure 3F—5 mg · kg: Time; *F*_12,48_ = 7.241, *p <* .0001: Treatment; *F*_1,48_ = 32.79, *p <* .0001: Interaction; *F*_12,48_ = 6.940, *p <* .0001, 20 min: *p* = .0004, 30 min: *p* < .0001, 40 min: *p* < .0001, 50 min: *p* = .0143).

### 3.4 Time of day elicits differential CORT response to ketamine infusion

To identify if time of day influences the responsiveness of the HPA axis, we compared the peak CORT responses to ketamine during the active and inactive phases. We found during the inactive phase, ketamine increased maximal CORT in a dose dependent manner (Figure 4A—*F*_3,12_ = 78.89, *p <* .0001: 0 vs. 5: *p* = .0182; 0 vs. 10: *p* = .0055; 0 vs. 25: *p* < .0001). Interestingly, this was not evident across any dose during the active period (Figure 4B—*F*_3,13_ = 1.489, *p* = .2637: 0 vs. 5: *p* = .2374; 0 vs. 10: *p* = .3205; 0 vs. 25: *p* = .9339), suggesting that not only the dose, but the timing of ketamine infusion may be important. Toward this, we found that the peak CORT concentration was only different with ketamine (25 mg · kg) when comparing active and inactive period data (Figure 4C—Treatment; *F*_3,14_ = 16.71, *p <* .0001: Interaction; *F*_3,11_ = 19.24, *p =* .0001, 25 mg · kg: *p* = .0001). However, to delineate the action of ketamine on the CORT response, we needed to remove the underlying circadian regulation of CORT secretion. Here, we measured the concentration of CORT as a percentage of time‐matched baseline and found that only when ketamine was infused during the inactive phase did it significantly increase the CORT response (Figure 4D— Time; *F*_1,25_ = 216.6, *p <* .0001: Treatment; *F*_3,25_ = 85.53, *p <* .0001: Interaction; *F*_3,25_ = 85.74, *p <* .0001: 5 mg · kg: *p* = .0025; 10 mg · kg: *p* = .0005; 25 mg · kg: *p* < .0001). This implies ketamine's effects on the HPA axis and GR activity may only be actionable if infused during the inactive period, when circulating endogenous glucocorticoids are low.

## 4 DISCUSSION

In this study, we investigated HPA axis activity in response to ketamine treatment at different times of day in an endogenous model of depression. In over 30% of clinically depressed patients, antidepressants are ineffective.^26^, ^27^, ^28^ However, ketamine, an anesthetic since 1970, has rapid onset antidepressant effects in a cohort of treatment‐resistant patients,^29^, ^30^ yet its actions on HPA axis function are not fully resolved.

The HPA axis is a neurohormonal system that utilizes feed‐forward and feedback loops to regulate glucocorticoid hormone levels. Within the hypothalamus, the paraventricular nucleus is highly responsive to external physiological stimuli such as the light/dark cycle, owing to the classically observed circadian release of glucocorticoids in timing with the day cycle.^9^ In addition to this, glucocorticoids can be released from the adrenal gland rapidly, through sympathetic splanchnic nerve stimulation, often a result of acute stress. It is well established that glucocorticoids mediate the body's response to stress.^9^ CORT is the most common biomarker studied in depression, with evidence suggesting that depression is associated with attenuated circadian variation of cortisol, as well as hypercortisolemia.^12^, ^31^, ^32^ The WKY rat—initially developed as a control for the spontaneous hypersensitive rat—has been used as a model of depression due to its hyper‐responsiveness to stress,^16^ yet the circadian variation and endogenous basal levels of corticosterone have not been determined.

We found no distinct differences in circadian release of CORT in the WKY strain (low in the inactive period, high in the active period). However, when we compared the levels to that of the commonly used SD rat, we saw a trend to an overall increase in circulating corticosterone. It is surprising that we did not find the reported increased baseline levels of corticosterone, however we employed an automated blood sampling system that allowed for blood collection from undisturbed rats.^24^, ^33^ This suggests that the significant increase in CORT levels previously seen may be due to hyperactivity of the HPA axis when handling and/or blood sampling. That being said, studies have reported altered density of GRs in the WKY rat^34^, ^35^ and decreased GR function contributes to HPA axis hyperactivity and the development of depressive symptoms,^36^ which may account for the depressive‐like phenotype seen in these rats.

Studies have investigated the interaction of ketamine with the HPA axis,^23^, ^37^ and although ketamine is regularly used to treat depression,^38^ the importance of timing—something critical to HPA axis function—has been overlooked. Ketamine acts as a rapid clinical antidepressant, as fast as 30 min following injection at 0.5 mg · kg in humans (10 mg · kg in rat).^39^, ^40^, ^41^ Yet whether the responsiveness of classical depression biomarkers such as CORT and HPA axis function differs across time is unknown. Studies suggest antidepressants modulate the GR.^36^, ^37^ For instance, the therapeutic action of antidepressants can restore GR function.^42^

Importantly, stressors can stimulate the sympathetic nervous system to rapidly activate the HPA axis, release noradrenaline from the locus coeruleus, and increase heart rate.^43^, ^44^ A large body of work has studied the effects of ketamine on the sympathetic nervous system,^45^, ^46^ suggesting that ketamine's action on corticosterone secretion may also be mediated via sympathetic nervous action, although in these studies anaesthetic doses of ketamine were used. Dissociating the actions of subanesthetic doses of ketamine on sympathetic action and its influence on HPA axis function warrants further investigation.

In this study, using repeated blood sampling in undisturbed, freely behaving rats, we report robust secretion of corticosterone in rats infused with subanesthetic ketamine doses. Interestingly, our findings support the response being a direct result of HPA axis activity, rather than sympathetic action on adrenal activity as our data show a clear delay in CORT response—with a significant increase first observed 20 min following infusion. However, in our study, ketamine is delivered subcutaneously, and the delay in HPA axis response may be the result of a delay in ketamine reaching circulation. Yet, the differential time of day CORT response suggests this is not the case.

Crucially, our data suggests that the timing of ketamine administration may be critical to HPA axis activity. We found that with equivalent doses of ketamine, corticosterone levels increased substantially if ketamine was administered during the inactive period. During this period, glucocorticoids are not routinely bound to the receptor. Several studies report that depressed patients have GR resistance (i.e., reduced GR function).^36^, ^47^, ^48^ Indeed, most antidepressants increase GR‐mediated gene transcription and GR function, which in turn is associated with enhanced GR‐mediated negative feedback by glucocorticoids.^47^, ^49^ Therefore, the sudden increase in circulating CORT during the inactive period (or when circulating levels are low) may act beneficially to bind to GRs to mediate negative feedback inhibition of the HPA axis to maintain low glucocorticoid levels under normal physiological conditions.

In summary, interactions between cortisol and ketamine have been reported,^23^, ^50^, ^51^ yet these studies measure responses over short periods of time, and potentially miss long term activity of the HPA axis. In this study, we found that the HPA axis response to ketamine was far stronger when ketamine was administered during the inactive period. These findings suggest that the timing of ketamine administration may be an important factor to consider when evaluating treatments for depression.

## AUTHOR CONTRIBUTIONS

**Matthew Birnie:** Conceptualization; data curation; formal analysis; methodology; writing – original draft; writing – review and editing. **Alen V. Eapen:** Data curation. **Yvonne Kershaw:** Data curation. **David Lodge:** Conceptualization; methodology. **Graham Collingridge:** Conceptualization; methodology. **Becky Conway‐Campbell:** Conceptualization; funding acquisition; investigation; methodology; project administration; supervision; writing – review and editing. **Stafford Louis Lightman:** Conceptualization; funding acquisition; investigation; methodology; project administration; supervision; writing – review and editing.

## CONFLICT OF INTEREST

The authors declare no competing interests.

### PEER REVIEW

The peer review history for this article is available at https://publons.com/publon/10.1111/jne.13194.

## Data Availability

Raw data were generated at the University of Bristol, UK. Derived data supporting the findings of this study are available from the corresponding author [MTB] on request." cd_value_code="text

## References

[1] Ferrari AJ , Charlson FJ , Norman RE , et al. Burden of depressive disorders by country, sex, age, and year: findings from the global burden of disease study 2010. PLoS Med. 2013;10:e1001547. 24223526 10.1371/journal.pmed.1001547 PMC3818162

[2] Nutt DJ . Relationship of neurotransmitters to the symptoms of major depressive disorder. J Clin Psychiatry. 2008;61:4‐7. 18494537

[3] Godlewska BR , Norbury R , Selvaraj S , Cowen PJ , Harmer CJ . Short‐term SSRI treatment normalises amygdala hyperactivity in depressed patients. Psychol Med. 2012;42:2609‐2617. 22716999 10.1017/S0033291712000591 PMC3488813

[4] Yoshimura R , Hori H , Ikenouchi‐Sugita A , Umene‐Nakano W , Ueda N , Nakamura J . Higher plasma interleukin‐6 (IL‐6) level is associated with SSRI‐ or SNRI‐refractory depression. Prog Neuropsychopharmacol Biol Psychiatry. 2009;33:722‐726. 19332097 10.1016/j.pnpbp.2009.03.020

[5] Papakostas GI , Nutt DJ , Hallett LA , Tucker VL , Krishen A , Fava M . Resolution of sleepiness and fatigue in major depressive disorder: a comparison of bupropion and the selective serotonin reuptake inhibitors. Biol Psychiatry. 2006;60:1350‐1355. 16934768 10.1016/j.biopsych.2006.06.015

[6] Rush AJ , Thase ME , Dubé S . Research issues in the study of difficult‐to‐treat depression. Biol Psychiatry. 2003;53:743‐753. 12706958 10.1016/s0006-3223(03)00088-x

[7] Voineskos D , Daskalakis ZJ , Blumberger DM . Management of treatment‐resistant depression: challenges and strategies. Neuropsychiatr Dis Treat. 2020;16:221‐234. 32021216 10.2147/NDT.S198774 PMC6982454

[8] Lang UE , Borgwardt S . Molecular mechanisms of depression: perspectives on new treatment strategies. Cell Physiol Biochem. 2013;31:761‐777. 23735822 10.1159/000350094

[9] Lightman SL , Birnie MT , Conway‐Campbell BL . Dynamics of ACTH and cortisol secretion and implications for disease. Endocr Rev. 2020;41:470‐490. 10.1210/endrev/bnaa002 PMC7240781 32060528

[10] Shea A , Walsh C , MacMillan H , Steiner M . Child maltreatment and HPA axis dysregulation: relationship to major depressive disorder and post traumatic stress disorder in females. Psychoneuroendocrinology. 2005;30:162‐178. 15471614 10.1016/j.psyneuen.2004.07.001

[11] Aihara M , Ida I , Yuuki N , et al. HPA axis dysfunction in unmedicated major depressive disorder and its normalization by pharmacotherapy correlates with alteration of neural activity in prefrontal cortex and limbic/paralimbic regions. Psychiatry Res. 2007;155:245‐256. 17587554 10.1016/j.pscychresns.2006.11.002

[12] Pariante CM , Lightman SL . The HPA axis in major depression: classical theories and new developments. Trends Neurosci. 2008;31:464‐468. 18675469 10.1016/j.tins.2008.06.006

[13] Overstreet DH . Modeling depression in animal models. Methods Mol Biol. 2012;829:125‐144. 22231810 10.1007/978-1-61779-458-2_7

[14] Paré WP . The performance of WKY rats on three tests of emotional behavior. Physiol Behav. 1992;51:1051‐1056. 1615043 10.1016/0031-9384(92)90091-f

[15] Paré WP . Open field, learned helplessness, conditioned defensive burying, and forced‐swim tests in WKY rats. Physiol Behav. 1994;55:433‐439. 8190758 10.1016/0031-9384(94)90097-3

[16] Solberg LC , Olson SL , Turek FW , Redei E . Altered hormone levels and circadian rhythm of activity in the WKY rat, a putative animal model of depression. Am J Physiol Regul Integr Comp Physiol. 2001;281:R786‐R794. 11506993 10.1152/ajpregu.2001.281.3.R786

[17] López‐Rubalcava C , Lucki I . Strain differences in the behavioral effects of antidepressant drugs in the rat forced swimming test. Neuropsychopharmacology. 2000;22:191‐199. 10649831 10.1016/S0893-133X(99)00100-1

[18] Kohrs R , Durieux ME . Ketamine: teaching an old drug new tricks. Anesth Analg. 1998;87:1186‐1193. 9806706 10.1097/00000539-199811000-00039

[19] Aan Het Rot M , Zarate CA , Charney DS , Mathew SJ . Ketamine for depression: where do we go from here? Biol Psychiatry. 2012;72:537‐547. 22705040 10.1016/j.biopsych.2012.05.003 PMC3438349

[20] Aan Het Rot M , Collins KA , Murrough JW , et al. Safety and efficacy of repeated‐dose intravenous ketamine for treatment‐resistant depression. Biol Psychiatry. 2010;67:139‐145. 19897179 10.1016/j.biopsych.2009.08.038

[21] Phillips JL , Norris S , Talbot J , et al. Single, repeated, and maintenance ketamine infusions for treatment‐resistant depression: a randomized controlled trial. Am J Psychiatry. 2019;176:401‐409. 30922101 10.1176/appi.ajp.2018.18070834

[22] Juven‐Wetzler A , Cohen H , Kaplan Z , Kohen A , Porat O , Zohar J . Immediate ketamine treatment does not prevent posttraumatic stress responses in an animal model for PTSD. Eur Neuropsychopharmacol. 2014;24:469‐479. 24239430 10.1016/j.euroneuro.2013.08.007

[23] Khalili‐Mahani N , Martini CH , Olofsen E , Dahan A , Niesters M . Effect of subanaesthetic ketamine on plasma and saliva cortisol secretion. Br J Anaesth. 2015;115:68‐75. 25982133 10.1093/bja/aev135

[24] Waite EJ , McKenna M , Kershaw Y , et al. Ultradian corticosterone secretion is maintained in the absence of circadian cues. Eur J Neurosci. 2012;36:3142‐3150. 22823558 10.1111/j.1460-9568.2012.08213.x

[25] Martinez‐Lozano Sinues P , Kohler M , Brown SA , Zenobi R , Dallmann R . Gauging circadian variation in ketamine metabolism by real‐time breath analysis. Chem Commun. 2017;53:2264‐2267. 10.1039/c6cc09061c 28150005

[26] Nassir GS . Why antidepressants are not antidepressants: STEP‐BD, STAR*D, and the return of neurotic depression. Bipolar Disord. 2008;10:957‐968. 19594510 10.1111/j.1399-5618.2008.00639.x

[27] Barbui C , Cipriani A , Patel V , Ayuso‐Mateos JL , van Ommeren M . Efficacy of antidepressants and benzodiazepines in minor depression: systematic review and meta‐analysis. Br J Psychiatry. 2011;198:11‐16. 21200071 10.1192/bjp.bp.109.076448 PMC3014462

[28] Fournier JC , DeRubeis RJ , Hollon SD , et al. Antidepressant drug effects and depression severity: a patient‐level meta‐analysis. JAMA. 2010;303:47‐53. 20051569 10.1001/jama.2009.1943 PMC3712503

[29] Serafini G , Howland R , Rovedi F , Girardi P , Amore M . The role of ketamine in treatment‐resistant depression: a systematic review. Curr Neuropharmacol. 2014;12:444‐461. 25426012 10.2174/1570159X12666140619204251 PMC4243034

[30] Schwartz J , Murrough JW , Iosifescu DV . Ketamine for treatment‐resistant depression: recent developments and clinical applications. Evid Based Ment Health. 2016;19:35‐38. 27053196 10.1136/eb-2016-102355 PMC10699412

[31] Keller J , Flores B , Gomez RG , et al. Cortisol circadian rhythm alterations in psychotic major depression. Biol Psychiatry. 2006;60:275‐281. 16458262 10.1016/j.biopsych.2005.10.014

[32] Gillespie CF , Nemeroff CB . Hypercortisolemia and depression. Psychosom Med. 2005;67:S26‐S28. 15953796 10.1097/01.psy.0000163456.22154.d2

[33] Flynn BP , Birnie MT , Kershaw YM , et al. Corticosterone pattern‐dependent glucocorticoid receptor binding and transcriptional regulation within the liver. PLoS Genet. 2021;17:e1009737. 34375333 10.1371/journal.pgen.1009737 PMC8378686

[34] Panarelli M , Holloway CD , Barr ABP , Fraser R , Kenyon CJ . Differences in temperature‐sensitive receptor binding of glucocorticoids in spontaneously hypertensive and normotensive Wistar‐Kyoto rats. Steroids. 1995;60:73‐75. 7792820 10.1016/0039-128x(94)00011-z

[35] Mileva GR , Moyes C , Syed S , Bielajew C . Strain differences and effects of environmental manipulation on astrocytes (glial fibrillary acidic protein), glucocorticoid receptor, and microglia (Iba1) immunoreactivity between Wistar‐Kyoto and Wistar females. Neuropsychobiology. 2017;75:1‐11. 28700991 10.1159/000476035

[36] Pariante CM , Miller AH . Glucocorticoid receptors in major depression: relevance to pathophysiology and treatment. Biol Psychiatry. 2001;49:391‐404. 11274650 10.1016/s0006-3223(00)01088-x

[37] Wang W , Liu L , Yang X , et al. Ketamine improved depressive‐like behaviors via hippocampal glucocorticoid receptor in chronic stress induced‐ susceptible mice. Behav Brain Res. 2019;364:75‐84. 30753876 10.1016/j.bbr.2019.01.057

[38] McIntyre RS , Rosenblat JD , Nemeroff CB , et al. Synthesizing the evidence for ketamine and esketamine in treatment‐resistant depression: an international expert opinion on the available evidence and implementation. Am J Psychiatry. 2021;178:383‐399. 33726522 10.1176/appi.ajp.2020.20081251 PMC9635017

[39] Fava M , Freeman MP , Flynn M , et al. Double‐blind, placebo‐controlled, dose‐ranging trial of intravenous ketamine as adjunctive therapy in treatment‐resistant depression (TRD). Mol Psychiatry. 2020;25:1592‐1603. 30283029 10.1038/s41380-018-0256-5 PMC6447473

[40] Carlson PJ , Diazgranados N , Nugent AC , et al. Neural correlates of rapid antidepressant response to ketamine in treatment‐resistant unipolar depression: a preliminary positron emission tomography study. Biol Psychiatry. 2013;73:1213‐1221. 23540908 10.1016/j.biopsych.2013.02.008 PMC3672258

[41] Radford KD , Park TY , Jaiswal S , et al. Enhanced fear memories and brain glucose metabolism (18F‐FDG‐PET) following sub‐anesthetic intravenous ketamine infusion in Sprague‐Dawley rats. Transl Psychiatry. 2018;8:263. 30504810 10.1038/s41398-018-0310-8 PMC6269482

[42] Budziñski ML , Sokn C , Gobbini R , et al. Tricyclic antidepressants target FKBP51 SUMOylation to restore glucocorticoid receptor activity. Mol Psychiatry. 2022;27:2533‐2545. 10.1038/s41380-022-01491-0 35256747

[43] Porges SW . Cardiac vagal tone: a physiological index of stress. Neurosci Biobehav Rev. 1995;19:225‐233. 7630578 10.1016/0149-7634(94)00066-a

[44] Curtis AL , Lechner SM , Pavcovich LA , Valentino RJ . Activation of the locus coeruleus noradrenergic system by intracoerulear microinfusion of corticotropin‐releasing factor: effects on discharge rate, cortical norepinephrine levels and cortical electroencephalographic activity. J Pharmacol Exp Ther. 1997;281:163‐172. 9103494

[45] Goddard K , Sampson C , Bedy S‐M , Ghadban R , Stilley J . Effect of ketamine on cardiovascular function during procedural sedation of adults. Cureus. 2021;13:e14228. 10.7759/cureus.14228 33948417 PMC8087490

[46] Komatsu T , Singh PK , Kimura T , Nishiwaki K , Bando K , Shimada Y . Differential effects of ketamine and midazolam on heart rate variability. Can J Anaesth. 1995;42:1003‐1009. 8590488 10.1007/BF03011073

[47] Pariante CM , Makoff A , Lovestone S , et al. Antidepressants enhance glucocorticoid receptor function in vitro by modulating the membrane steroid transporters. Br J Pharmacol. 2001;134:1335‐1343. 11704655 10.1038/sj.bjp.0704368 PMC1573058

[48] D'Alessio L , Mesarosova L , Anink JJ , et al. Reduced expression of the glucocorticoid receptor in the hippocampus of patients with drug‐resistant temporal lobe epilepsy and comorbid depression. Epilepsia. 2020;61:1595‐1605. 32652588 10.1111/epi.16598 PMC7496961

[49] Arana GW , Santos AB , Laraia MT , et al. Dexamethasone for the treatment of depression: a randomized, placebo‐ controlled, double‐blind trial. Am J Psychiatry. 1995;152:265‐267. 7840362 10.1176/ajp.152.2.265

[50] Zhao Y , Xie W , Dai J , Wang Z , Huang Y . The varying effects of short‐term and long‐term corticosterone injections on depression‐like behavior in mice. Brain Res. 2009;1261:82‐90. 19401166 10.1016/j.brainres.2008.12.083

[51] Camargo A , Dalmagro AP , Wolin IAV , Siteneski A , Zeni ALB , Rodrigues ALS . A low‐dose combination of ketamine and guanosine counteracts corticosterone‐induced depressive‐like behavior and hippocampal synaptic impairments via mTORC1 signaling. Prog Neuropsychopharmacol Biol Psychiatry. 2021;111:110371. 34089815 10.1016/j.pnpbp.2021.110371

